# Femtosecond Laser Microfabrication of Ti_3_C_2_T_*x*_ MXene for Micro-Supercapacitor Electrodes

**DOI:** 10.1021/acsomega.5c08652

**Published:** 2026-01-20

**Authors:** Kelly T. Paula, Murilo H. M. Facure, Marcelo. B. Andrade, Daniel S. Correa, Cleber R. Mendonca

**Affiliations:** † Universidade de Sao Paulo, Instituto de Física de São Carlos, São Carlos, SP 3560-970, Brazil; ‡ Embrapa Instrumentacao, Nanotechnology National Laboratory for Agriculture (LNNA), São Carlos 13560-970, Brazil; § Universidade Federal de Sao Carlos, Departamento de Química, São Carlos, SP 13565-905, Brazil; ∥ Universidade Federal de Ouro Preto, Departamento de Física, Ouro Preto, MG 35402-136, Brazil

## Abstract

There is growing interest in using MXene-based materials for electronic and energy storage applications by integrating advanced microfabrication techniques. In this work, we investigate the femtosecond laser micromachining of Ti_3_C_2_T*_x_* MXene films to enable the direct fabrication of microsupercapacitor (MSC) electrodes with high precision and minimal thermal damage. The influence of pulse energy and number of pulses on the resulting microstructures was systematically analyzed using Scanning Electron Microscopy, Atomic Force Microscopy, Energy Dispersive X-ray Spectroscopy, and Raman spectroscopy. Irradiation with low pulse counts (1–5 pulses at 1010 nJ) produced localized features with depths of 0.5–1.0 μm and minimal redeposition, whereas higher pulse numbers (up to 20,000) yielded features of ∼1.2 μm with partial material removal and resolidified regions. Incubation analysis revealed a progressive reduction in ablation threshold with increasing pulse number. Elemental mapping and Raman spectra confirmed efficient material removal and exposure of the underlying substrate. Using optimized parameters, interdigitated electrodes were fabricated and integrated into planar MSCs, which exhibited an areal capacitance of 19 mF/cm^2^ at 5 mV/s, as well as energy and power densities of 0.45 μWh/cm^2^ and 0.3 mW/cm^2^ at 1 mA/cm^2^, respectively. These results demonstrate that femtosecond laser processing provides a versatile and high-resolution approach for MXene patterning, with strong potential for scalable microdevice fabrication in energy-related technologies.

## Graphical abstract

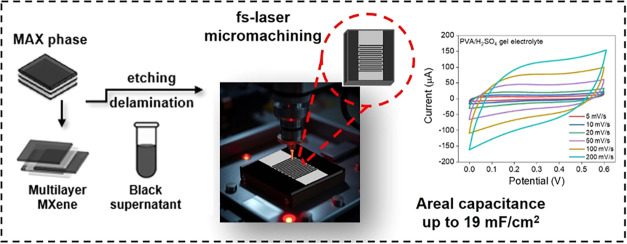

## 1 Introduction

MXenes are a class of two-dimensional transition metal carbides, nitrides, and carbonitrides that have gained significant attention due to their exceptional physical and chemical properties.^1,2^ Their high electrical conductivity, excellent chemical stability, large specific surface area, and hydrophilic nature, combined with tunable surface terminations, make them particularly attractive for applications in energy storage.^3,4^ These characteristics facilitate rapid ion transport and high charge storage capacity, while offering compatibility with a wide range of processing techniques.^5,6^ Owing to this unique combination of features, MXenes have emerged as highly promising materials for developing advanced electrochemical devices, especially microsupercapacitors (MSCs), where efficient charge collection and fast electrochemical response are essential.^7^

The production of devices at the microscale depends critically on developing precise, efficient, and scalable fabrication techniques.^8^ Conventional microfabrication methods, including photolithography and chemical etching, often involve complex multistep processes, chemical agents that can degrade the material, and limited flexibility for prototyping and rapid patterning.^9,10^ In this context, femtosecond (fs) laser micromachining emerges as a powerful tool for direct patterning functional materials.^11–13^ Due to the extremely short pulse duration, on the order of 10^–15^ s, energy is deposited on the material faster than it can thermally diffuse, enabling nonthermal ablation with minimal thermal damage.^14^ This ultrafast energy delivery not only preserves the intrinsic properties of sensitive materials, such as MXenes, but also allows micromachining with submicron resolution. Unlike other conventional microfabrication techniques, femtosecond laser processing is a maskless and chemical etching-free single-step method that offers direct, rapid, and high-resolution structuring of materials.^15,16^ These characteristics make it especially attractive for applications requiring precise material removal or modification, such as in the fabrication of microfluidic,^17^ photonic,^18,19^ biomedical^20^ and microelectromechanical systems.^21^ Owing to these features, femtosecond laser processing has been explored for structuring various functional materials, enabling high-precision microfabrication of energy storage devices.^22,23^ The ultrafast nature of femtosecond laser pulses allows for controlled ablation, phase transformations, and surface modifications at the microscale.^24,25^

In recent years, fs-laser processing has been explored for micro/nanostructuring a variety of functional thin films, including graphene,^26^ transition metal dichalcogenides,^27^ and carbon-based materials.^28,29^ For MXenes, fs-laser micromachining has emerged as a promising strategy, although several challenges remain. Reported issues include the tendency of MXenes to oxidize under laser irradiation, difficulties in achieving uniform and residue-free ablation, and the risk of impairing their electrochemical performance due to thermal or chemical side effects.^30–32^ Prior studies have applied fs-laser ablation to pattern Ti_3_C_2_T*_x_* films for device fabrication, including high-rate supercapacitors^33^ and terahertz polarizers,^34^ establishing important groundwork on MXene–laser interactions. In this study, the emphasis shifts to understanding how laser-processing parameters influence the morphological, chemical, and electrochemical evolution of amorphous Ti_3_C_2_T*_x_* during fs-LIFT-based microfabrication. This perspective highlights the material transformation mechanisms induced by the transfer process, offering a complementary view to direct-ablation approaches. Further systematic investigations remain essential to establish clear correlations between processing conditions (e.g., pulse number, fluence) and the resulting structural definition, stability, and electrochemical behavior of MXene films.

This work focuses on the systematic investigation of fs-laser micromachining to process MXene thin films, particularly Ti_3_C_2_T_*x*_ MXene, owing to its excellent electrochemical properties. A key aspect of the study is the evaluation of laser processing parameters, such as pulse energy and number of pulses, and their influence on the morphology and quality of micromachined features. By identifying the optimal laser parameters that enable clean and controlled ablation of the MXene surface, achieving complete material removal with minimal damage and well-defined features, it was possible to fabricate high-precision interdigitated electrode (IDE) structures directly on the MXene film. These IDEs were then used to assemble an MSC, demonstrating the practical application of the optimized fs-laser processing approach.

## 2 Experimental Procedure

### 2.1 Materials

The titanium aluminum carbide (Ti_3_AlC_2_, ≥90%, ≤200 μm) was obtained from Sigma-Aldrich. Hydrochloric acid (HCl, 36%, Synth) and lithium chloride (LiCl, Synth) were purchased from Synth, and hydrofluoric acid (HF, 40%) was purchased from Vetec. The poly­(vinyl alcohol) (PVA, 99+%, 89,000–98,000 g/mol) and the sulfuric acid (H_2_SO_4_, 98%) used in the electrolyte preparation were obtained from Sigma-Aldrich and Synth, respectively.

### 2.2 Ti_3_C_2_T_*x*_ MXene Synthesis

MXene was synthesized using an etching method of the MAX phase (Ti_3_AlC_2_), as described elsewhere.^35^ In brief, 2.4 mL of HF and 12 mL of HCl were added to 5.6 mL of water in a poly­(tetrafluoroethylene) (PTFE) container. Then, 1 g of Ti_3_AlC_2_ was slowly added to the solution, which was left stirring for 24 h at 35 °C for etching the A layer of the MAX phase. The resulting multilayer MXene was washed through repetitive centrifugation cycles (3500 rpm for 5 min) until the pH of the supernatant was higher than 6. To obtain the delaminated Ti_3_C_2_T_*x*_, the multilayered MXene was dispersed in a LiCl solution (50 mL, 0.5 mol/L) and stirred at 35 °C for 20 h. Next, centrifugation cycles (3500 rpm for 5 min) were initially used to remove the LiCl until the supernatant started to present a blackish color. After that, the black supernatant containing the delaminated MXene was collected. Considering the etching efficiency and delamination rate, the procedure yields approximately 75% of MXene relative to the initial MAX phase amount. A schematic representation of the MXene synthesis process is shown in Figure 1a.

**Figure 1:**
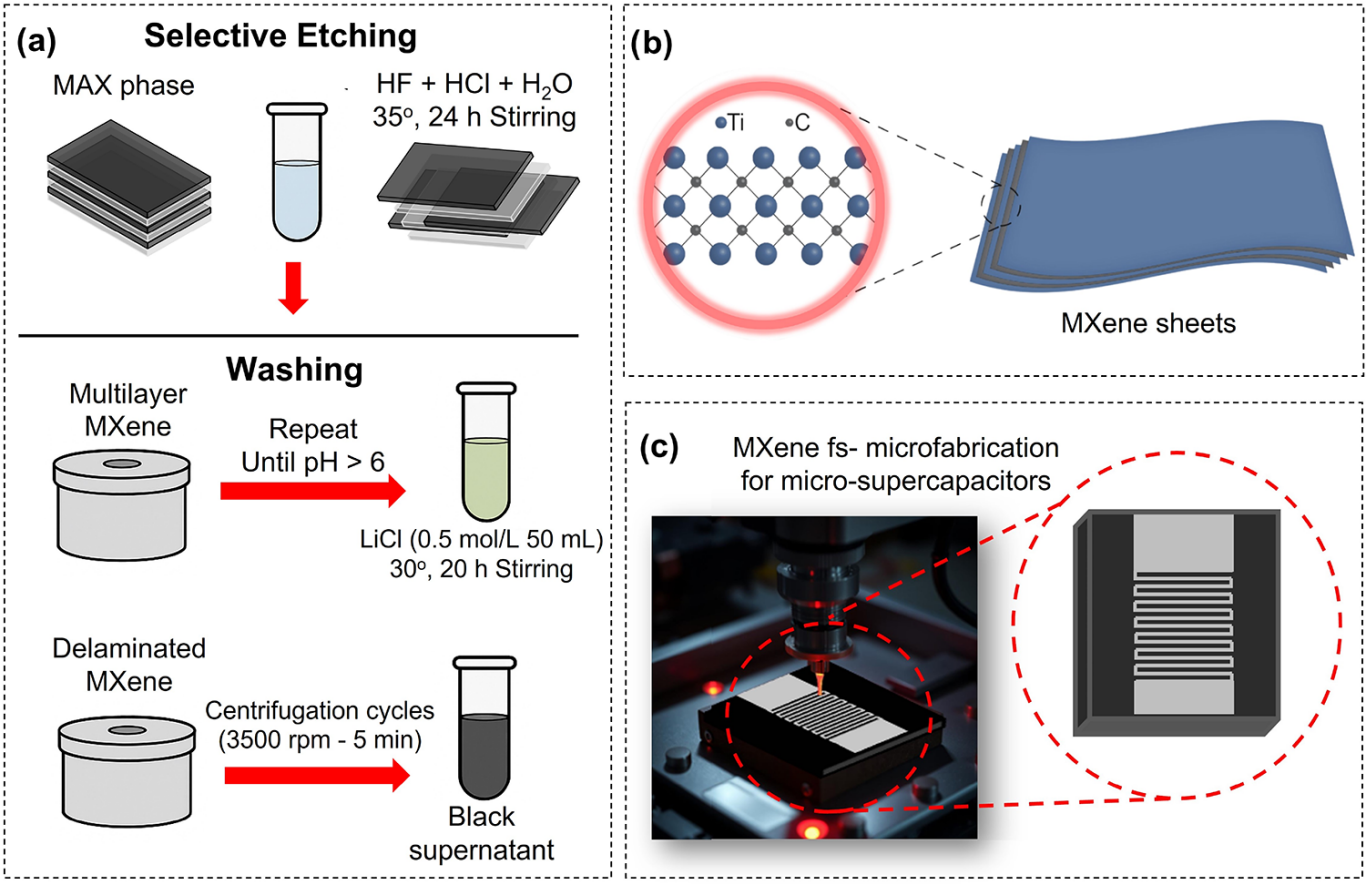
(a) Schematic representation of the MXene synthesis process, highlighting the main steps. (b) Representation of the MXene sheets. (c) Schematic representation of the femtosecond laser microfabrication process used to pattern interdigitated electrodes on a Ti_3_C_2_T_*x*_ MXene film.

To obtain the Ti_3_C_2_T_*x*_ MXene films, 400 μL of a 3.3 mg/mL dispersion of the delaminated Ti_3_C_2_T_*x*_, represented in Figure 2b, was drop-casted onto a glass slide (1 cm × 1 cm). After deposition, the films were allowed to dry overnight under ambient conditions to ensure uniform solvent evaporation and film formation. Film thickness was measured using AFM at several distinct points across the sample surface. These measurements consistently yielded thickness values within a narrow range, resulting in an average thickness of 1.2 ± 0.1 μm.

**Figure 2:**
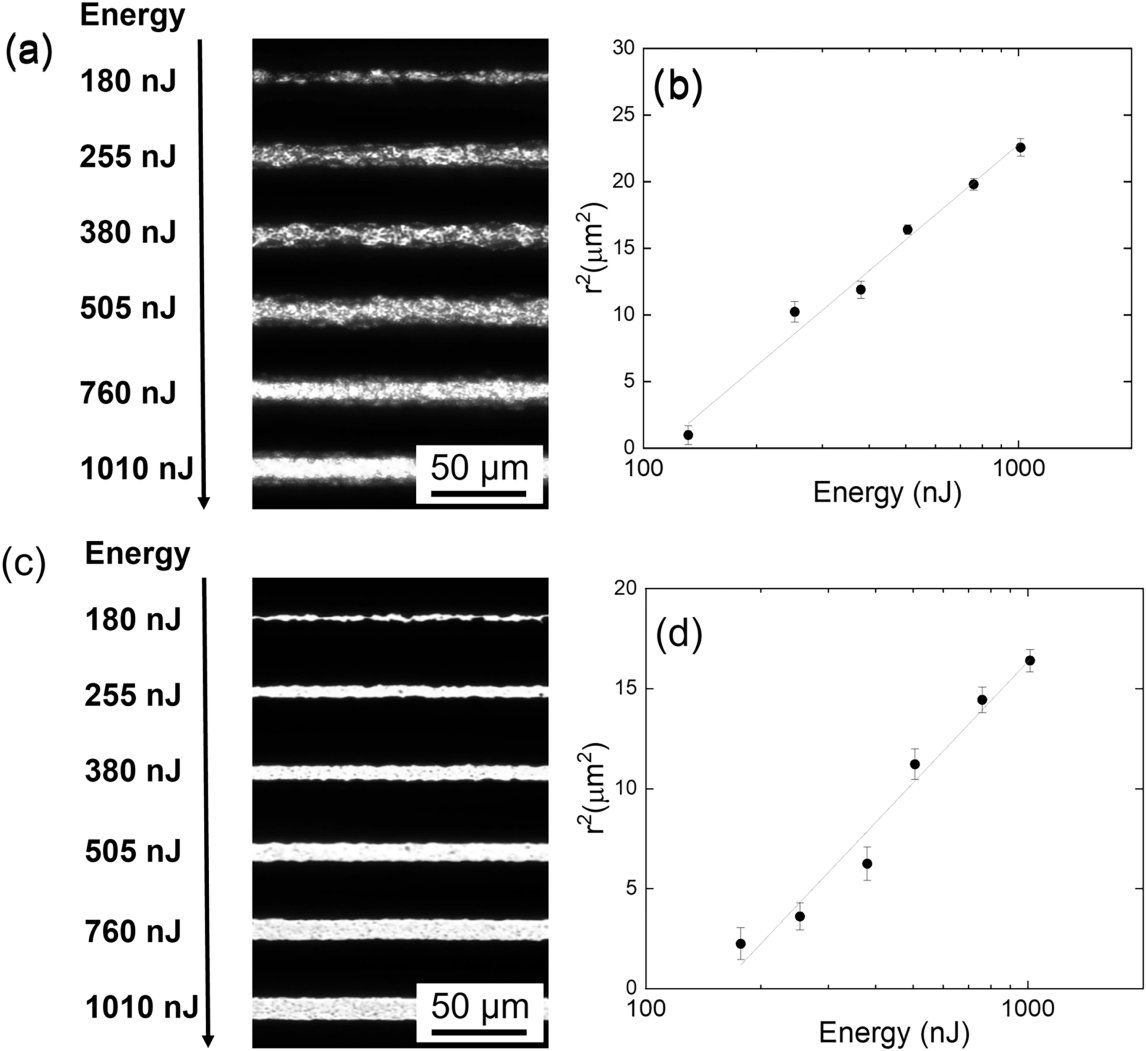
(a) Optical microscopy images of micromachined structures fabricated on the Ti_3_C_2_T_*x*_ MXene sample using *N* = 20,000 pulses and pulse energies ranging from 130 to 1010 nJ. (b) The squared line radius as a function of pulse energy for *N* = 20,000 pulses. (c) Optical microscopy images of micromachined structures fabricated on the Ti_3_C_2_T_*x*_ MXene sample using *N* = 5 pulses and pulse energies ranging from 180 to 1010 nJ. (d) The squared line radius as a function of pulse energy for *N* = 5 pulses.

### 2.3 Ti_3_C_2_T_*x*_ MXene Microfabrication

The microfabrication of MXene films was carried out using a diode-pumped Yb:KGW femtosecond laser system, which delivers ultrashort laser pulses of 216 fs duration at a central wavelength of 1,030 nm. The system allows for an adjustable repetition rate ranging from 20 Hz to 200 kHz, providing flexibility in tailoring the laser-matter interaction regime. The laser beam was tightly focused onto the sample surface using a microscope objective with a numerical aperture (NA) of 0.25, enabling high spatial resolution and efficient energy deposition required for precise micromachining.

To facilitate accurate and reproducible fabrication, the Ti_3_C_2_T_*x*_ MXene films were positioned on a motorized three-axis (*xyz*) translational stage with submicron resolution. The motorized stage allowed for controlled sample translation at constant speeds, generating a wide range of microstructures with well-defined geometries. The patterning process was monitored in real-time using a CCD camera aligned coaxially with the optical axis of the focusing objective, allowing visual feedback of the ablation process and alignment precision throughout the structuring process.

A systematic investigation of laser parameters was performed to optimize the fabrication process. The pulse energy was varied from 180 nJ to 1530 nJ, while the scanning speed ranged from 12.5 to 25.0 μm/s. These parameters were carefully chosen to explore their effects on the ablation threshold, feature dimensions, and structural integrity of the Ti_3_C_2_T_*x*_ MXene films. In particular, the relationship between laser fluence and the resulting microstructure morphology was analyzed to identify the optimal conditions for clean material removal without damaging the underlying substrate.

The number of laser pulses (*N*) was controlled by adjusting both the sample translation speed (*v*) and the laser repetition rate (*f*). The number of pulses per spot is given by $N≅1.25\frac{fw_{0}}{v}$ , in which *w*_0_ is the beam waist. For each selected number of pulses *N*, the single-pulse energy was systematically varied to obtain the structure radius as a function of energy. This procedure enabled the determination of the ablation threshold for each *N*, thereby allowing us to evaluate the incubation effect on MXene films.

An interdigitated electrode architecture was fabricated using the optimized laser parameters, resulting in uniform and well-defined microstructured features suitable for electrochemical applications. This design was selected to maximize the active surface area, reduce ion diffusion distances, and enhance charge collection efficiency. The high-precision control of the *xyz* translational stage ensured accurate fabrication of the electrode geometry, contributing to the reproducibility and performance of the resulting MSC, as illustrated in Figure 1c.

### 2.4 Characterization Techniques

The laser-fabricated Ti_3_C_2_T_*x*_ MXene microstructures were extensively characterized using complementary analytical techniques. Surface morphology and elemental composition were investigated through Scanning Electron Microscopy (SEM) and Energy Dispersive X-ray Spectroscopy (EDX), both performed using a TM3000-Hitachi microscope equipped with a Bruker Quantax EDX system. Topographic features at the nanoscale were evaluated using Atomic Force Microscopy (AFM) with a Nanosurf easyScan 2 system. Raman spectroscopy was performed to investigate the structural and vibrational characteristics of the samples, utilizing a LabRAM HR Evolution confocal micro-Raman spectrometer equipped with a liquid nitrogen-cooled CCD detector. A 532 nm excitation laser was employed using a 100× objective (NA = 0.9) to achieve high spatial resolution. Together, these techniques enabled a detailed investigation of the morphological and structural characteristics of the laser-processed Ti_3_C_2_T*_x_* MXene films.

### 2.5 Electrochemical Measurements

The electrochemical tests were performed using a potentiostat/galvanostat PGSTAT30 Autolab (Metrohm). All measurements were conducted using a PVA/H_2_SO_4_ gel electrolyte prepared as reported previously.^36^ Initially, a 10 wt % PVA gel was prepared by dissolving 1 g of PVA in 10 mL of water at 90 °C under continuous stirring for 4 h, resulting in a homogeneous transparent gel. Then, 0.84 mL of H_2_SO_4_ was added to the PVA gel, which mixture was then stirred for 1 h to obtain the 1 M PVA/H_2_SO_4_ gel electrolyte.

The cyclic voltammetry (CV) experiments were carried out over a potential range from 0 to 0.6 V at different scan rates, ranging from 5 mV/s to 200 mV/s. The galvanostatic charge–discharge (GCD) curves were obtained at current densities of 0.5 mA/cm^2^ and 1 mA/cm^2^.

The areal capacitance (*C*_A_) of the MXene MSC was calculated using

1 $$C_{A}=\int \frac{idt}{\mathrm{AV}}$$

where *i* is the current measured as a function of time (*t*), *V* is the voltage window, and *A* is the effective area of the MXene MSC. The energy (*E*_A_) and power (*P*_A_) densities were calculated using, respectively

2 $$E_{A}=\frac{C_{A}V^{2}}{2}$$

3 $$P_{A}=\frac{E_{A}}{\Delta t}$$

in which Δ*t* is the discharge time obtained from the GCD curve.

## 3 Results and Discussion

Figure 2 shows optical microscopy images of micromachined structures produced on the Ti_3_C_2_T_*x*_ MXene sample using femtosecond laser processing under varying experimental conditions. The micromachining was performed using a focused fs-laser beam with different pulse energies and scanning speeds to investigate the influence of laser parameters on the resulting surface modifications. The images in Figure 2 reveal distinct patterns formed on the Ti_3_C_2_T_*x*_ MXene surface, demonstrating the effect of pulse energy, and number of pulses on the material’s response to laser exposure. The formation of micromachined lines, with widths of a few micrometers, indicates a precise and controlled material removal process enabled by the ultrafast nature of the fs-laser pulses.

Figure 2a presents optical microscopy images depicting micromachined lines produced with *N* = 20,000 pulses and pulse energies ranging from 130 to 1010 nJ. In comparison, Figure 2c displays optical images of micromachined lines using *N* = 5 pulses and pulse energies ranging from 180 to 1010 nJ. The half-line width (*r*) was extracted from the optical microscopy images for each set of conditions. The corresponding plots of *r* ^2^ as a function of pulse energy (*E*) are displayed in Figure 2b,d, representing the Ti_3_C_2_T_*x*_ MXene sample processed with *N* = 20,000 and *N* = 5 pulses, respectively. For these specific data sets, *r* ranged from 1 to 4.75 μm and from about 1 to 3.6 μm for lines microstructured using *N* = 20,000 and *N* = 5 pulses, respectively. Across a broader range of experiments conducted on the Ti_3_C_2_T_*x*_ MXene sample, with variations in the number of pulses, the average radius varied from around 1 to 5 μm, while *N* varied from 20,000 to 1 pulse.

Considering the Gaussian spatial distribution of the laser beam employed in the micromachining process, the damage threshold energy (*E*_th_) was determined using the zero-damage method.^37^ By fitting the data presented in Figure 2b,2d (solid lines), threshold energies values of 109.8 nJ and 163.8 nJ were determined, respectively, when using *N* = 20000 and *N* = 5 pulses. Additionally, from the same fittings, the beam radius at the focus (*w*_0_) was estimated to be 4.3 μm. Using these values, the corresponding threshold laser fluence (*F*_th_) were calculated to be 0.36 and 1.27 J/cm^2^, respectively. These findings contribute to a better understanding of femtosecond laser processing of Ti_3_C_2_T_*x*_ MXene, providing valuable insights for optimizing laser parameters for applications in microelectronics and sensing technologies.

Figure 3a presents scanning electron microscopy (SEM) images of micromachined structures fabricated on the MXene sample using a pulse energy of 1010 nJ for different numbers of pulses. These images provide insights into the resolution and surface morphology of the laser-processed regions, highlighting the influence of pulse accumulation on material modification. As the number of pulses increases, progressive structural changes are observed, indicating variations in material removal efficiency and defect accumulation. To address potential thermal effects during multipulse irradiation, it is important to note that femtosecond ablation of Ti_3_C_2_T_*x*_ is dominated by nonthermal mechanisms under single-pulse excitation. However, when operating at kHz repetition rates and high pulse overlap, partial heat accumulation may occur. This contribution can influence the morphology of the laser-modified regions, particularly at the highest values of pulse superposition used here, and was taken into account in the interpretation of the ablation features.

**Figure 3:**
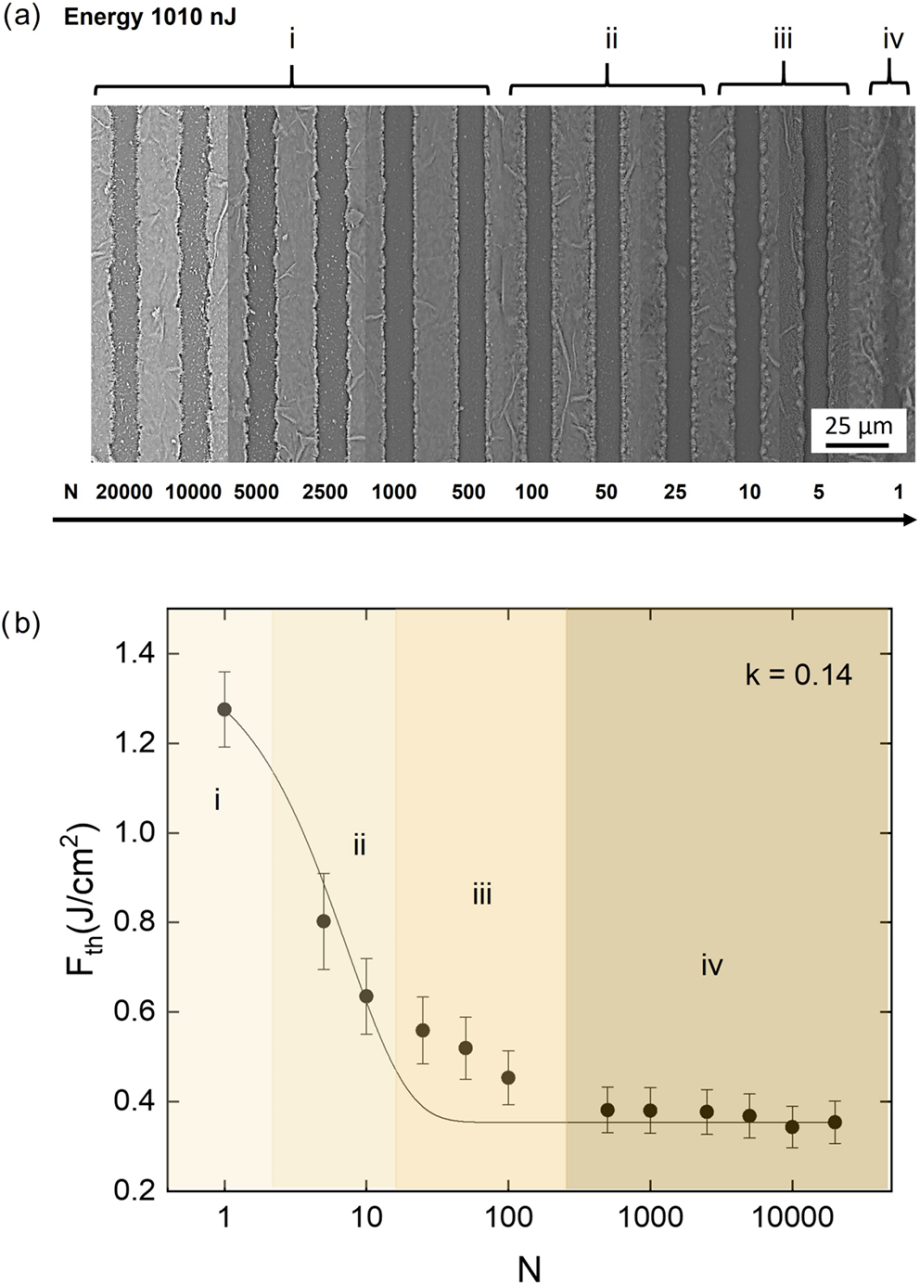
(a) Scanning electron microscopy images of micromachined structures on the Ti_3_C_2_T_*x*_ MXene sample, fabricated using a pulse energy of 1010 nJ with varying numbers of pulses. (b) Incubation curve depicting the relationship between the threshold fluence (*F*_th_) and the number of laser pulses (*N*) applied to the Ti_3_C_2_T_*x*_ MXene sample.

Figure 3b displays the incubation curve, illustrating the relationship between the threshold fluence and the number of laser pulses applied to the Ti_3_C_2_T_*x*_ MXene sample. A key observation is that the energy threshold required to induce damage decreases with an increasing number of pulses, a phenomenon attributed to the incubation effect. This effect, widely studied in various materials, including semiconductors,^38^ metals,^39^ and ceramics,^40^ plays a critical role in determining laser processing parameters for precise material structuring.

During laser irradiation, each pulse deposits energy into the material, generating defects such as vacancies and dislocations. These defects act as precursors for subsequent laser-induced modifications, gradually lowering the energy required to achieve ablation.^41^ Initially, the defect density remains relatively low at a low pulse count, and the energy threshold for material removal is high. As the pulse count increases, however, the accumulation of defects enhances the material’s sensitivity to laser exposure, reducing the threshold fluence.^42^ This decrease stabilizes beyond a certain number of pulses, indicating a saturation point where defect accumulation no longer significantly influences the threshold energy.

To further elucidate the mechanism of defect accumulation observed in this work, it is important to consider the role of heat accumulation in ultrafast laser–material interactions. As reported in studies on ultrafast laser processing, the interaction proceeds through nonlinear absorption and transient carrier excitation, followed by localized thermalization.^43^ When the pulse count and repetition rate increase, successive pulses deposit energy before complete thermal relaxation, thereby enhancing the local lattice temperature and reducing the fluence threshold for material modification. This heat accumulation effect explains the observed increase in ablation efficiency and morphological changes with higher pulse numbers, and is consistent with mechanisms described for ultrafast laser.^43^ Such effects are particularly relevant for femtosecond laser microfabrication of Ti_3_C_2_T_*x*_ MXene, where controlled defect engineering and precise structuring depend critically on the interplay between nonlinear absorption, thermal accumulation, and material-specific thermo-physical properties.

The incubation effect observed in Figure 3b can be quantitatively described by the exponential defect accumulation model, which provides a more accurate representation of our experimental findings. The threshold fluence after irradiation with *N* pulses (*F*_th,_ *_N_*) is related to the single-pulse threshold fluence (*F*_th,1_) and the asymptotic fluence threshold (*F*_th,∞_) through the expression^44^

4 $$F_{\mathrm{th},N}=\left(F_{\mathrm{th},1}-F_{\mathrm{th},\infty }\right)e^{-k\left(N-1\right)}+F_{\mathrm{th},\infty }$$

where *k* represents the incubation parameter.

The incubation behavior of Ti_3_C_2_T_*x*_ MXene was analyzed using the defect accumulation model, yielding an incubation parameter of *k* = 0.14 ± 0.02. This value was obtained from the fitting of experimental incubation curves, considering the single-pulse threshold (*F*_th,1_ = 0.82 J/cm^2^) and the asymptotic threshold (*F*_th,∞_ = 0.35 J/cm^2^). The incubation parameter indicates a relatively efficient accumulation of modifications with successive laser pulses, leading to a quicker transition to the ablation regime. When compared with other materials, the obtained value places Ti_3_C_2_T_*x*_ in an intermediate regime of defect accumulation. For example, graphene has been reported to have *k* ≈ 0.91,^45^ and TiAlN/TiN multilayer coatings present *k* ≈ 0.642,^46^ while diamond-like carbon exhibits a much lower value, around 0.004,^47^ reflecting its higher structural resilience.

These comparisons suggest that Ti_3_C_2_T_*x*_ MXene films exhibit a relatively low incubation parameter, pointing to a more efficient accumulation of subthreshold modifications and a faster transition to the ablation regime. This behavior can be associated with the unique layered morphology and chemically active surface terminations of Ti_3_C_2_T_*x*_ MXenes, which enhance their responsiveness to femtosecond laser pulses and make them suitable for precision microfabrication applications.

The incubation curve resulting from femtosecond laser micromachining of Ti_3_C_2_T_*x*_ MXene exhibits a reduction in the threshold fluence as the number of pulses increases, eventually reaching a saturation point. To better interpret this behavior, we have divided the incubation curve into four distinct regions (Figure 3), each associated with a specific structuring process. In region (i), corresponding to the initial exposure, the material requires a relatively high threshold fluence to induce damage. As the number of pulses increases within the region (ii), the threshold fluence begins to decrease due to the accumulation of defects and structural modifications within the material. Moving into the region (iii), this trend continues, with a further decline in threshold fluence as the laser-induced defects accumulate and facilitate subsequent material modification. Finally, in region (iv), the incubation effect reaches saturation, indicating that the threshold fluence stabilizes despite the increasing number of pulses. The decrease in threshold fluence occurs over a range of approximately 1 to 100 pulses, stabilizing at 0,35 J/cm^2^. This trend suggests that the Ti_3_C_2_ T_*x*_ MXene material exhibits a relatively low incubation parameter, indicating that a significant number of pulses is required to induce structural modifications. These results reinforce the applicability of the exponential defect accumulation model,^44^ supporting that laser-generated defects progressively alter the material’s response to subsequent irradiation.

Figure 4a displays AFM micrographs of micromachined lines on the Ti_3_C_2_T_*x*_ MXene sample. These images were captured within a 40 × 40 μm^2^ region of the micromachined structures, using a pulse energy of 1010 nJ and varying the number of pulses between 10,000, 100, 5, and 1. A 3D AFM image was also obtained for *N* = 5 and is illustrated in Figure 4c.

**Figure 4:**
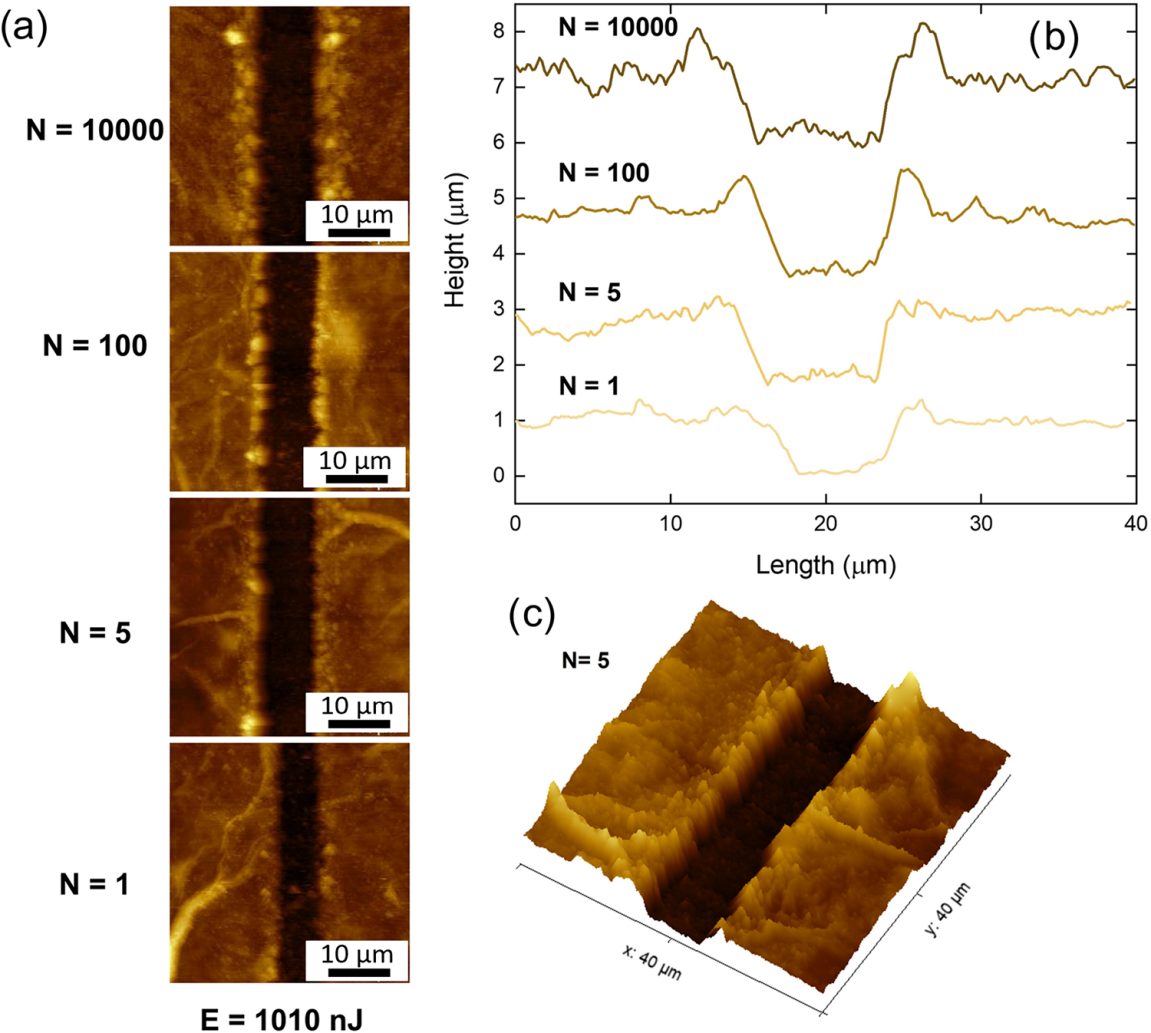
(a) Atomic force microscopy micrographs of micromachined lines on the Ti_3_C_2_T_*x*_ MXene sample, using a pulse energy of 1010 nJ and varying pulse numbers (10,000, 100, 5, and 1). (b) AFM profiles illustrating the depth variations of the micromachined lines as a function of pulse number (*N*). (c) 3D AFM image of a micromachined line produced with *N* = 5, providing a topographical view of the laser-induced surface features.

The AFM profiles corresponding to different pulse numbers are analyzed in Figure 4b. The micromachining process consistently demonstrates a correlation between pulse energy and the resulting structural features. As the pulse energy increases, the height and depth of the machined lines also increase. This trend is observed across all tested pulse energy levels and pulse counts, highlighting the direct relationship between laser parameters and the morphology of microfabricated structures. A comparative evaluation of the micromachined lines further reveals that variations in pulse number significantly influence the depth and shape of the structures. A higher number of pulses does not necessarily result in complete material removal; instead, it can lead to surface modification and partial redeposition. Conversely, fewer pulses promote more effective material removal, producing well-defined features. These observations are consistent with broader trends reported in the literature for ultrafast laser micro/nanostructuring, where careful tuning of irradiation parameters enables precise control of feature geometry and depth.^48^ For example, in recent studies on laser-optical-field-modulation for fabricating large-aperture dual-band MWIR/LWIR antireflection windows, variations in laser exposure conditions directly modulated structural depth and shape to achieve the desired optical performance.^49^

By incorporating the incubation effect analysis, the laser-induced structures, shown in Figure 4, can be categorized into four distinct regions based on the number of pulses and resulting morphological features. Region IV (20,000 to 500 pulses) exhibits ablation depths ranging from 1.0 to 1.2 μm as the pulse energy increases from 130 to 1010 nJ. In this regime, thermal accumulation leads to the formation of resolidified material along the edges and center of the ablated regions. Region III (100 to 25 pulses) shows minimal variation in depth, with similar edge features, though the resolidified material is less prominent, indicating a gradual reduction in thermal effects. In Region II (10 to 5 pulses), the depths range from 0.8 to 1.2 μm across the same energy window, but edge resolidification becomes negligible, suggesting a transition toward more efficient and cleaner ablation. Finally, in Region I (single-pulse regime), precise and highly localized structures are formed with depths of up to 1.0 μm at high energies (1520 nJ), and as low as 0.5 μm at reduced energies (430 nJ), with no evidence of resolidified material, confirming the occurrence of ultrafast, nonthermal ablation.

These observations reinforce the influence of pulse number on the microstructural characteristics of the Ti_3_C_2_T_*x*_ MXene sample. As the number of pulses decreases, the material removal process becomes more efficient, minimizing redeposition effects and yielding structures with well-defined profiles.

To gain a more detailed understanding of the chemical composition of the microstructures produced by femtosecond laser processing, EDX mapping was conducted to identify the main elements present on the surface. Figure 5 presents the results of this analysis for the structure obtained using 5 pulses and an energy of 1010 nJ. Specifically, Figure 5a shows the elemental mapping of titanium (Ti), while Figure 5b displays the silicon (Si) distribution. As expected, the results indicate a decrease in Ti concentration in the modified region, while the presence of Si increases. This behavior is attributed to the ablation process, in which the surface material, primarily composed of Ti, is removed, exposing the underlying silicon substrate. Figure 5c illustrates a line scan, where the Ti and Si concentrations variation along the microstructure was analyzed. The blue line represents the Ti profile, while the green line corresponds to Si. It is observed that the Ti signal intensity decreases in the micromachined region, while Si exhibits a significant increase, confirming the selective removal of surface material. These results are consistent with the spectroscopic data shown in Figure 5d, which indicate a reduction in the intensity of the characteristic Ti_3_C_2_T_*x*_ MXene peaks after laser irradiation. The combination of EDX mapping and Raman spectroscopy confirms the effectiveness of the femtosecond laser micromachining process in the controlled material removal, allowing for the fabrication of well-defined microstructures and revealing the chemical composition of the laser-modified regions.

**Figure 5:**
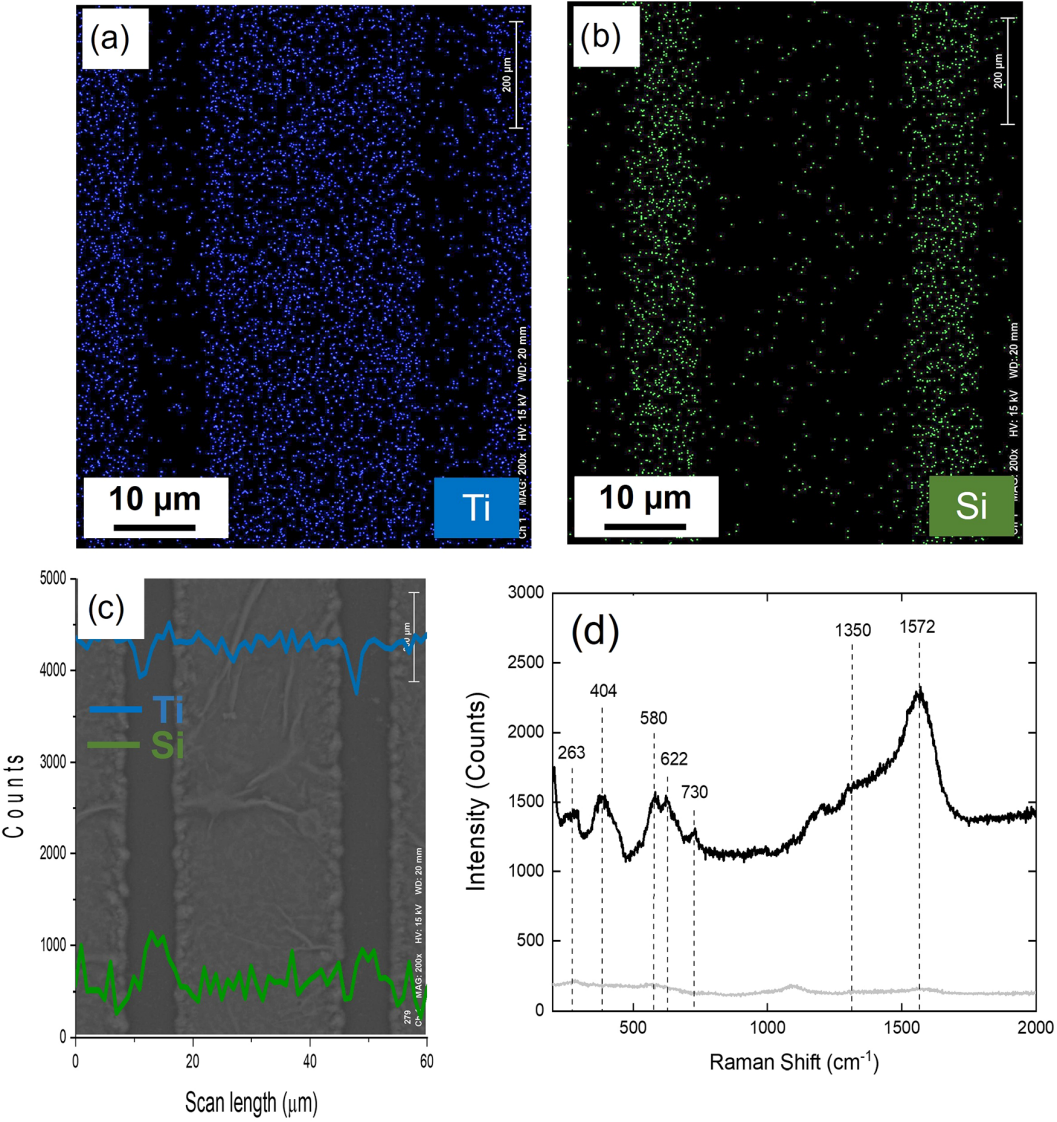
(a) EDX elemental mapping of titanium (Ti) on the laser-micromachined Ti_3_C_2_T_*x*_ MXene sample. (b) EDX mapping of silicon (Si). (c) Line scan analysis of Ti (blue) and Si (green) concentrations along the microstructure. (d) Raman spectra of the Ti_3_C_2_T_*x*_ MXene sample before (black) and after (red) femtosecond laser irradiation.

Raman spectroscopy analysis provides critical insights into the materials’ structural and chemical composition, making it a valuable tool for assessing modifications induced by laser micromachining. In Figure 5d, the Raman spectra of the Ti_3_C_2_T_*x*_ MXene sample before and after femtosecond laser irradiation are presented, allowing for the evaluation of structural integrity and material removal effectiveness. The spectrum of the unirradiated region (black line) exhibits well-defined peaks characteristic of Ti_3_C_2_, including vibrational modes at 286, 404, 580, 622, and 725 cm^–1^. The peaks at 286 and 404 cm^–1^ correspond to E_g_ vibrational modes related to the titanium surface groups,^50,51^ while the A_1g_ peak at 580 cm^–1^, together with the E_g_ and A_1g_ peaks at 622 and 725 cm^–1^, respectively, are attributed to carbon vibrations within the Ti_3_C_2_T_*x*_ MXene structure.^52^ Additionally, two broad peaks appear in the 1000–1800 cm^–1^ range, associated with graphitic carbon.^53^ Specifically, the D-band at approximately 1350 cm^–1^ is linked to the A_1g_ vibrational mode,^53,54^ indicative of structural disorder and defects in sp^2^ carbon rings, while the G-band at 1572 cm^–1^, corresponding to the E_2g_ vibrational mode,^53,54^ represents the stretching of C–C bonds in sp^2^-hybridized carbon structures. The presence of these peaks confirms the structural integrity of the Ti_3_C_2_T_*x*_ MXene before laser processing.

In contrast, the Raman spectrum of the laser-irradiated region displays a substantial reduction in peak intensities, suggesting effective material removal due to the ablation process. The suppression of characteristic Ti_3_C_2_T_*x*_ MXene peaks indicates that femtosecond laser micromachining successfully alters the material, reducing its Raman-active features and confirming the efficiency of laser-induced ablation. This result corroborates the capability of laser-based fabrication in inducing structural changes in MXenes, providing essential information for optimizing the conditions for the development of applications.

To demonstrate the potential of this approach for the development of MXene-based MSC, interdigitated electrodes (IDEs) were precisely fabricated by femtosecond laser micromachining. The resulting IDEs comprised 50 interdigitated fingers, each approximately 3 mm long and with a uniform width close to 10 μm, separated by 50 μm gaps. This electrode architecture was chosen to ensure a large surface area-to-volume ratio, short ion diffusion paths, and efficient charge collection, which features are essential for achieving high performance in planar energy storage devices.^55^

Optimal laser parameters for the fabrication process were determined to be 5 pulses and an energy of 1010 nJ, which enabled complete removal of the Ti_3_C_2_T_*x*_ MXene material in the patterned regions and resulted in well-defined, uniform digit features. As mentioned previously, Figure 1c presents a schematic illustration of the femtosecond laser microfabrication process, showing the laser beam being focused onto the Ti_3_C_2_T_*x*_ MXene film through a microscope objective to induce localized ablation. The diagram emphasizes the fabrication of interdigitated electrodes with well-defined features directly on the film surface. Figure 6a displays a real image of the fabricated MSC, highlighting the interdigitated electrode architecture. An optical microscopy inset provides a closer view of the digit structures, confirming the precision and uniformity achieved through laser micromachining.

**Figure 6:**
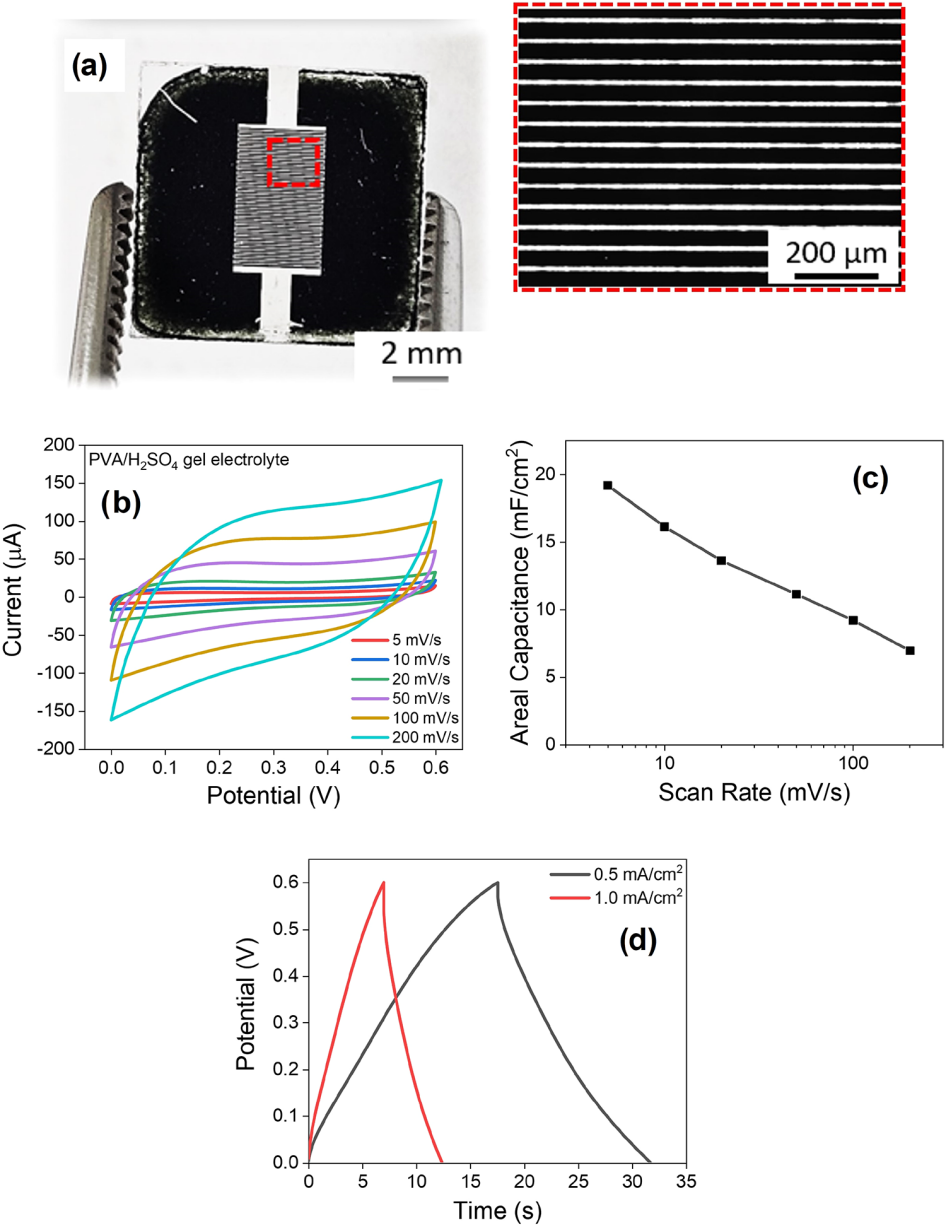
(a) Photograph of the fabricated Ti_3_C_2_T_*x*_ MXene microsupercapacitor (MSC), produced by femtosecond laser microfabrication, with an optical microscopy image highlighting the interdigitated electrode structure. (b) Cyclic voltammetry (CV) curves obtained at scan rates ranging from 5 to 200 mV/s. (c) Areal capacitance as a function of scan rate. (d) Galvanostatic charge–discharge (GCD) curves at different current densities.

As a proof of concept, the symmetric MSCs were tested in a two-electrode configuration using a 1 M PVA/H_2_SO_4_ gel electrolyte. As shown in Figure 6b, nearly rectangular cyclic voltammograms were obtained at scan rates ranging from 5 mV/s to 200 mV/s. This behavior is consistent with previous reports on Ti_3_C_2_T_*x*_-based MSC^56,57^ and indicates the capacitive nature of the device.^58^ The fabricated Ti_3_C_2_T_*x*_ MSC exhibited an areal capacitance of 19 mF/cm^2^ at a scan rate of 5 mV/s, a value comparable to others Ti_3_C_2_T_*x*_ MSC reported in the literature.^59–61^ As the scan rate increased up to 200 mV/s, the areal capacitance decreased nearly linearly, reaching 7 mF/cm^2^, as illustrated in Figure 6c.

Figure 6d displays the GCD curves for the obtained MSC at current densities of 0.5 mA/cm^2^ and 1 mA/cm^2^. In agreement with the CV results, the GCD curves show a linear voltage–time relationship during charging and discharging, further confirming the purely capacitive behavior of the device.^62^ The MXene-based microsupercapacitor demonstrated Coulombic efficiencies of 80.2% and 77.0% at current densities of 0.5 mA/cm^2^ and 1 mA/cm^2^, respectively. The asymmetry of the GCD curves can be attributed to the large substrate surface area combined with the limited thickness of the active material film.^63^ The device presented energy and power densities of 0.45 μWh/cm^2^ and 0.3 mW/cm^2^ at 1 mA/cm^2^, and 0.6 μWh/cm^2^ and 0.15 mW/cm^2^ at 0.5 mA/cm^2^, respectively. These values are in line with those reported for other MXene-based microsupercapacitors. For example, an in situ annealed Ti_3_C_2_T_*x*_ device delivered areal energy densities up to 6.94–7.53 μWh cm^–2^ with corresponding power densities of 0.20–0.90 mW/cm^2^.^64^ Similarly, MXene–graphene composite aerogel 3D MSCs exhibited an energy density of 2.18 μWh cm^–2^ at a power density of 60 μW cm^–2^.^65^ In addition, clay-like Ti_3_C_2_T_*x*_ MXene MSCs achieved 0.77 μWh cm^–2^ at a high power density of 46.6 mW cm^–2^.^66^ Compared with these reports, the devices fabricated in this work exhibit competitive performance, confirming the viability of femtosecond laser processing for efficient MXene microsupercapacitors. Although the referenced studies employed different fabrication techniques, often based on conventional lithography, printing, or masking approaches, the femtosecond laser microfabrication used here presents notable advantages. These include high spatial resolution, maskless and direct-write processing, rapid prototyping capability, and greater design freedom for creating arbitrary patterns and scalable architectures.

In practical applications, interdigitated electrodes (IDEs) must satisfy specific processing requirements, including well-defined line widths and interspaces, generally in the micrometer range, high pattern uniformity, smooth ablation profiles, and reproducibility over large areas. These parameters directly affect ion diffusion lengths, electrode surface area, and ultimately the electrochemical performance of MSCs. The fs-laser micromachining approach used in this work fulfills these requirements by producing IDEs with controlled widths of ∼10 μm and interspaces of ∼50 μm, uniform geometry, and minimal redeposition. These characteristics are consistent with the practical specifications reported for advanced MSC devices, demonstrating that the structures fabricated here are suitable for real applications.

Such attributes make the proposed method competitive in terms of performance and significantly more versatile and efficient for the rapid development of advanced MXene-based microsupercapacitors. Moreover, the technique offers the potential for further optimization through modifications in the interdigitated electrode architecture and by integrating Ti_3_C_2_T_*x*_ MXene with other active materials to produce charge storage devices with improved performance.

## 4 Conclusions

This work demonstrates that femtosecond laser micromachining is an effective method for precisely patterning Ti_3_C_2_T_*x*_ MXene films, enabling the fabrication of high-performance microsupercapacitors (MSCs). Systematic variation of pulse energy and number revealed a clear dependence of ablation morphology on irradiation parameters. Low pulse counts (1–5 pulses) produced localized, clean features with minimal redeposition, while higher pulse numbers generated deeper structures with altered edge morphology. Incubation analysis confirmed a progressive reduction in ablation threshold with increasing pulse number, consistent with defect accumulation. Chemical and spectroscopic analyses verified the controlled removal of material and exposure of the substrate. Optimized processing (5 pulses at 1010 nJ) enabled the fabrication of interdigitated electrodes that exhibited promising electrochemical performance, with an areal capacitance of 19 mF/cm^2^ at 5 mV/s and energy/power densities of 0.45 μWh/cm^2^ and 0.3 mW/cm^2^ at 1 mA/cm^2^. These results establish femtosecond laser processing as a versatile, maskless approach for tunable microfabrication of MXene films, with potential for next-generation on-chip energy storage devices.
